# Production of active Exendin-4 in *Nicotiana benthamiana* and its application in treatment of type-2 diabetics

**DOI:** 10.3389/fpls.2022.1062658

**Published:** 2022-12-22

**Authors:** Shammi Akter, Shajia Afrin, Jaeyoon Kim, Joohyun Kang, Md Abdur Razzak, Per-Olof Berggren, Inhwan Hwang

**Affiliations:** ^1^ Department of Life Sciences, Pohang University of Science and Technology, Pohang, South Korea; ^2^ Department of Research and Development, BioN Inc., Pohang, South Korea; ^3^ The Rolf Luft Research Center for Diabetes and Endocrinology, Karolinska Institutet, Stockholm, Sweden

**Keywords:** Exendin-4, type-2 diabetics, enterokinase, insulin, recombinant protein production

## Abstract

GLP-1 (Glucagon-like peptide-1) is a peptide that stimulates insulin secretion from the β-cell for glycemic control of the plasma blood glucose level. Its mimetic exenatide (synthetic Exendin-4) with a longer half-life of approximately 3.3–4 h is widely used in clinical application to treat diabetes. Currently, exenatide is chemically synthesized. In this study, we report that the GLP-1 analogue recombinant Exendin-4 (Exdn-4) can be produced at a high level in *Nicotiana benthamiana*, with an estimated yield of 50.0 µg/g fresh biomass. For high-level expression, we generated a recombinant gene, *B:GB1:ddCBD1m:8xHis : Exendin-4* (*BGC : Exdn-4*), for the production of Exendin-4 using various domains such as the BiP signal peptide, the GB1 domain (B1 domain of streptococcal G protein), a double cellulose binding domain 1 (CBD1), and 8 His residues (8xHis) to the N-terminus of Exendin-4. GB1 was used to increase the expression, whereas double CBD1 and 8xHis were included as affinity tags for easy purification using MCC beads and Ni^2+^-NTA resin, respectively. BGC : Exdn-4 was purified by single-step purification to near homogeneity using both Ni^2+^-NTA resin and microcrystalline cellulose (MCC) beads. Moreover, Exdn-4 without any extra residues was produced from BGC : Exdn-4 bound onto MCC beads by treating with enterokinase. Plant-produced Exdn-4 (Exendin-4) was as effective as chemically synthesized Exendin-4 in glucose-induced insulin secretion (GIIS) from mouse MIN6m9 cells a pancreatic beta cell line.

## Introduction

Diabetes mellitus is one of the most common diseases worldwide. It is a serious health issue and also an economic burden on society. In 2021, diabetes affected around 537 million people aged 20–79. One estimation forecasted that the number is going to rise to 643 million by 2030 and 783 million by 2045 **(** www.diabetesatlas.org). Especially in low- and middle-income countries, 3 out of every 4 adults are thought to live with diabetes. Currently, diabetes mellitus is the third-largest cause of death in industrialized countries after cardiovascular disease and cancer (Barfoed, 1987). There are multiple types of diabetes, with type-2 diabetes being dominant. In type-2 diabetes patients, many complications arise, including cardiovascular disease and kidney disease (Zhang et al., 2010; Potenza et al., 2011). The main causes of type-2 diabetes are obesity, insulin resistance, inadequate insulin secretion from β-cells, and inappropriate glucagon secretion. As a result, a high glucose level in the blood leads to the development of hyperglycemia (Alonso-Magdalena et al., 2011). Although several diabetic medications are available in the market, many patients still have limited access as well as some side effects (Molitch, 2013).

There are several FDA-approved peptide drugs with high specificity and low toxicity as therapeutic agents for clinical application to diabetes. One of them is exenatide, which stimulates insulin secretion in type-2 diabetes patients (Bellmann-Sickert and Beck-Sickinger, 2010). Exenatide is a chemical synthetic form of a peptide hormone called Exendin-4 that occurs naturally in the saliva of the Gila monster, a large venomous lizard native to the southwestern United States and northwestern Mexico (Eng et al., 1992; Triplitt and Chiquette, 2006; Yap and Misuan, 2019) and is an analogue of glucagon-like peptide-1 (GLP-1). GLP-1 is a peptide hormone mainly secreted from intestinal L cells. It controls glycemia by acting as a glucose sensor, stimulates insulin secretion from β-cells, and suppresses glucagon release (Chia and Egan, 2005). In addition, it improves insulin sensitivity, slows gastric emptying, and reduces food intake, thereby reducing the blood glucose level (Mudaliar and Henry, 2012). However, naturally produced GLP-1 is rapidly degraded within 2 min by dipeptidyl peptidase-IV (DPP-IV) in blood. Exenatide (Byetta), synthetic Exendin-4, is resistant to DPP-IV (Young et al., 1999; Garber, 2011). Now, multiple forms of Exendin-4 have been developed, including immediate and extended-release forms.

Because of the growing number of patients with diabetes, there is a great demand for the production of Exendin-4. In this study, we investigated whether Exdn-4 can be produced in plants. Recently, plants have gained growing interest as a host of recombinant protein production. It has been proposed that plants could offer viable promising hosts for recombinant protein production with the premise of cost-effective, highly scalable, low-capital investment in the infrastructure; the ability to do post-translational modifications; and storage stability (Gupta et al., 2002). Furthermore, the unique advantage of a plant-based production system is safety because plant cells neither harbor human and animal pathogens nor produce endotoxins (Gerhartz, 1990; Daniell et al., 2001). Indeed, many pharmaceutical proteins have been successfully produced in plants. Taliglucerase α produced in carrot cells was introduced in the market to treat Gaucher disease (Fox, 2012; Zimran et al., 2018). Many growth factors or cytokines, such as human interleukin 6, human leukemia inhibitory factor (hLIF), epidermal growth factor, and human epidermal growth factor (hEGF), have been produced as active forms in plants (Hanittinan et al., 2020). In addition, CTB (Cholera toxin B subunit)-fused Exendin-4 has been produced in tobacco and lettuce chloroplasts for the purpose of oral delivery (Ruhlman et al., 2007; Kwon et al., 2013). However, one limitation is that the expression level of the gene of interest (GOI) by either transient gene expression or transgenic plants is still usually low. Therefore, high-level expression and efficient purification systems are urgently needed for the production of recombinant proteins in plants.

In this study, we aimed to produce Exdn-4 in *N. benthamiana via* the transient expression system. We generated a high-expression construct using various approaches including the use of a translational enhancing domain. In addition, we developed a cost-effective purification method to reduce the purification cost. Here, we present evidence that recombinant proteins containing Exendin-4 can be produced at high levels in *N. benthamiana* and easily purified by microcrystalline cellulose (MCC) beads or Ni^2+^-NTA resin. Moreover, active Exdn-4 without any extra residue can be produced from the recombinant proteins by proteolytic cleavage using enterokinase.

## Methods and materials

### Recombinant gene construction

A DNA fragment of 2L:EK : Exdn-4 consisting of 2 copies of GGGS as a linker, an enterokinase site, and Exendin-4 was chemically synthesized (Gene Universal, USA). 2L:EK : Exdn-4 was digested with *XmaI* and *XhoI* restriction endonucleases and ligated into the pTEX1::BiP : GB1:HL:GFP vector (Song et al., 2022), digested with the same endonucleases to give *BiP : GB1:HL:2L:EK : Exdn-4*. Subsequently, a DNA fragment, ddCBDm with 8xHis, that had been chemically synthesized (Gene Universal, USA) was digested with *BamH1* and *XmaI* restriction endonucleases and ligated into pTEX1::BiP : GB1:HL:2L:EK : Exdn-4 digested with *BamH1 and XmaI* restriction endonucleases to give pTEX1::B:G:ddCBDm : Exdn-4 (*BGC : Exdn-4*). To add the ER retention motif HDEL at the C-terminus of BGC : Exdn-4, PCR was performed using primers XmaI-2L-EK-F and XhoI-HDEL-R (**Supplementary Table 1**) and 2L:EK : Exdn-4 as template. The PCR product was digested with *XmaI* and *XhoI* restriction endonucleases and ligated to pTEX1::B:G:ddCBDm : Exdn-4 that had been digested with *XmaI* and *XhoI* restriction endonucleases to give pTEX1::B:G:ddCBDm : Exdn-4:HDEL (*BGC : Exdn-4:HDEL*).

### Plant materials and growth conditions

*Nicotiana benthamiana* plants were grown in a greenhouse under the conditions of 24°C, 40–65% relative humidity, and a long-day photoperiod (16 h light and 8 h dark, with illumination of 130–150 μmol m^-2^ sec^-1^) for 6–7 weeks. Six- to seven-week-old plants were used for agro-infiltration.

### Plasmid transformation into *Agrobacterium strain EHA105* & transient expression in *N. benthamiana*

For transient expression of the recombinant gene construct, *BGC : Exdn-4* & *BGC : Exdn-4:HDEL* in *N. benthamiana*, the binary expression vector was transformed into *Agrobacterium tumefaciens* strain EHA105 by electroporation, and transformed *Agrobacteria* were grown on LB plates supplemented with kanamycin (50 µg/mL) and rifampicin (50 µg/mL) at 28°C for 2 days. A single colony was grown in 5 mL of LB medium supplemented with kanamycin (50 µg/mL) and rifampicin (50 µg/mL) in a shaking incubator overnight. Then, 400 µl of overnight *Agrobacterium* culture was transferred to 50 mL of fresh LB medium supplemented with kanamycin (50 µg/mL) and rifampicin (50µg/mL). After growing for 16 h, the cells were centrifuged at 5,000 × g at 28°C for 10 min. The supernatant was discarded, and the pellet was resuspended in infiltration buffer (10 mM MES, and 10 mM MgSO_4_, pH 5.7). Finally, the *Agrobacterium* suspension was adjusted to 0.80 of OD_600_ by adding the infiltration buffer. Then, 400 µM acetosyringone was added to the *Agrobacterium* suspension, followed by incubation at room temperature for 1 h. *Agrobacterium* suspensions were infiltrated using a needleless syringe (Zhang et al., 2020). After infiltration, plants were further grown, and the leaf tissues were harvested on the 3^rd^, 5^th^, and 7^th^ days after infiltration (dpi).

For vacuum infiltration, the leaf tissues of *N. benthamiana* were submerged in *Agrobacterium* suspension in a vacuum chamber, and a 50–400 mbar vacuum was applied for 1 min. After the release of the vacuum, the plants were recovered from the vacuum chamber and washed with water to remove extra *Agrobacterium* suspension from leaf tissues. Plants were returned to the greenhouse and further grown for 5–7 days. Agroinfiltrated leaf tissue were harvested on 3 dpi, 5 dpi and 7 dpi (Buyel and Fischer, 2012).

### Protein extraction, SDS/PAGE,and western blot analysis

Leaf tissues were ground in liquid nitrogen using a mortar and pestle to a fine powder and mixed with 4 volumes (w/v) of protein extraction buffer (50 mM Tris-HCl, pH 7.5, 150 mM NaCl, 1 mM DTT, 0.1% [v/v] Triton X-100, and 1x protease inhibitor cocktail). The total soluble protein was recovered after centrifugation at 18,000 × g for 15 min. Protein concentration was measured using the Bradford protein assay (Bio-Rad, Hercules, CA, USA).

For Western blot analysis, 10 μg of total proteins were separated by SDS/PAGE. After SDS/PAGE, proteins were transferred onto PVDF membrane. The membrane with proteins was blocked with 6% non-fat dried milk in TBST buffer (20 mM Tris-HCl, pH 7.5, 500 mM NaCl, and 0.05% Tween-20) for 30 min to 1 h and incubated with anti-rabbit IgG antibody (1:5,000 dilution) at 4°C overnight. The membrane was washed three times with TBST buffer for 10 min each. Protein bands were detected by chemiluminescence (ECL; Amersham Pharmacia Biotech, Buckinghamshire, England), and images were obtained with an Amersham Imager 680 (AI680) analyzer.

### Purification of BGC : Exdn-4 using Ni^2+^-NTA resin and MCC beads

Leaf tissues (40 g fresh biomass) harvested at 5 dpi after *Agrobacterium*-mediated infiltration were ground to a fine powder with a mortar and pestle under liquid nitrogen. The powder was mixed with extraction buffer (50 mM Tris-HCl, pH 7.5, 150 mM NaCl, 0.1% Tween-20, 1X protease inhibitor cocktail, 1% PMSF) at 1:6 w/v, and the mixture was vortexed for 3–4 min. Total soluble protein extracts were centrifuged at 18,000 × g for 15 min, and the supernatant containing total soluble proteins was separately collected. To remove cellular debris as much as possible, the supernatant was centrifuged three additional times.

First, we purified BGC : Exdn-4 using Ni^2+^-NTA resin. To purify BGC : Exdn-4 using Ni^2+^-NTA resin, the column containing Ni^2+^-NTA resin (1 ml) was equilibrated with the extraction buffer. The total soluble protein extracts were adjusted to contain 350 mM NaCl and 8 mM imidazole at the final concentration. Total soluble protein extracts were loaded into the column and allowed to flow through the column by gravity. The solution that passed through the column was collected as the flow-through fraction. The resin in the column was washed with 150 mL of washing buffer (50 mM Tris-HCl, pH 7.5, 150 mM NaCl, 18 mM imidazole) five times, and the washing solution was collected as wash-off solution. The proteins bound to the resin were eluted using the elution buffer (50 mM Tris-HCl, pH 7.5, 150 mM NaCl, 400 mM imidazole). To ensure complete elution of BGC : Exdn-4, from the resin, proteins were eluted three times using 5 mL of the elution buffer each time. Immediately after elution, the elution buffer was changed with PBS buffer during a process of protein concentration using a Millipore 10 kD centrifugal filter (Millipore, Burlington, MA, United States). All steps were carried out at a flow rate of 0.5 mL/min.

To purify BGC : Exdn-4 proteins using microcrystalline cellulose (MCC) beads, total soluble protein extracts were mixed with MCC beads (Sigma-Aldrich, St. Louis, MO, USA) in a batch mode. Briefly, MCC beads (1 g) were suspended in four volumes of water, and the supernatant containing impurities was discarded. The washing of MCC beads was repeated five times. MCC beads were resuspended in water at a 1:1 ratio (v/v) (~4.1 mL bead volume). Thereafter, 240 mL TSP from 40 g fresh biomass was divided into six 50 mL centrifuge tubes (40 mL in each tube). Each tube was mixed with MCC slurry and placed at 4°C with gentle shaking for binding of proteins to the MCC beads. After incubation for 2 h, the MCC beads bound to proteins were collected by centrifugation at 3,000 × g for 2 min, and the supernatant (unbound fraction) and MCC beads with bound proteins were collected separately. The MCC beads were washed four times with four bead volumes of 40 mM Tris-HCl buffer (pH 7.5) to remove loosely bound proteins. To determine purity, proteins bound to MCC beads were released in 2× SDS/PAGE sample buffer by boiling for 10 min and analyzed by SDS/PAGE followed by CBB staining.

### Release of Exdn-4 from BGC : Exdn-4 by enterokinase

To obtain Exdn-4 without any extra amino acid residues, MCC beads bound to BGC : Exdn-4 were washed three times with 5 mL of 40 mM Tris-HCl (pH 7.4). Subsequently, the MCC beads were suspended in 5 mL of enterokinase cleavage buffer (20 mM Tris-HCl, pH 7.4, and 50 mM NaCl, 2 mM CaCl_2_). The mixture was incubated at 25°C for 12 h with gentle shaking. Released Exdn-4 was recovered in the supernatant after centrifugation at 3,000 × g at 4°C for 5 min. To recover any remaining Exdn-4 from the MCC beads, 2 mL of enterokinase cleavage buffer was added to the MCC beads, they were gently mixed for 1 min, and the supernatant was collected after centrifugation at 3,000 × g for 5 min. Exdn-4 was concentrated using a Millipore 3 kD filter (Millipore, Burlington, MA, United States) and stored at -80°C. For long-term storage (more than a week), Exdn-4 in PBS buffer was supplemented with 5% glycerol and stored at -80°C.

### Quantification and yield of BGC : Exdn-4

The amount of purified BGC : Exdn-4 was quantified by the Bradford protein assay using human serum albumin (HSA) as a protein standard. Alternatively, the amount of purified proteins was also quantified by comparing the intensity of Comassie brilliant blue (CBB)-stained Exdn-4 with that of HSA protein size standard bands on a 12% SDS/PAGE gel.

### Treatment of purified BGC : Exdn-4 with endo-H

Purified BCG : Exdn-4 (2 μg) was incubated with 1x glycoprotein denaturing buffer (0.5% SDS and 1 mM dithiothreitol) and denatured by boiling for 10 min. After this had cooled down to room temperature, 0.5 units of endo H (Roche, USA) were added to the denatured BGC : Exdn-4 and incubated at 37°C for 2 h, followed by analysis by Western blotting with anti-rabbit IgG antibody.

### Subcellular fraction of total protein extracts using a linear sucrose density gradient

The two constructs *BGC : Exdn-4* and *BGC : Exdn-4:HDEL* together with *GFP* were transiently expressed in *N. benthamiana via Agrobacterium*-mediated infiltration (Song et al., 2022). Total leaf tissues were harvested and homogenized using an active Motif Dounce Homogenizer (Thermofisher Catalog No.NC0569256) in 2 mL of homogenization buffer (50 mM Tris-HCl, pH 8.2, 20% [v/v] glycerol, 1 mM DTT, 1x protease inhibitor cocktail) per gram of tissue. Homogenates were filtered through Miracloth and centrifuged at 5,000 x g for 5 min. The supernatant was again centrifuged at 10,000 x g for 10 min. Subsequently, the supernatant was collected as total protein extracts and was mixed with 3 mL of homogenization buffer and filtered using 11 µM Nylon Net. The total protein extracts were further centrifuged at 5,000 x g for 10 min. 2 mL of the supernatant were loaded on the top of a linear sucrose density gradient (10% - 60% [w/w]) prepared in buffer (10 mM Tris-HCl, pH 7.6, and 1x protease inhibitors). After centrifugation in a swinging bucket rotor at 40,000 x g at 4°C for 18 h, 0.5-mL fractions were collected in a total of 22 fractions. Sucrose percentage of each fraction was measured using a refractometer (Fisher Scientific, Pittsburgh). Proteins in these fractions were separated by SDS-PAGE and analyzed by western blotting.

### RNA extraction and qRT-PCR

Agroinfiltrated leaf tissues were collected at 5 dpi and ground under liquid nitrogen using TissueLyser. The total RNA was purified using a Qiagen plant RNA purification kit (RNeasy Plant Mini Kit, Cat. No./ID: 74904), according to the protocol provided by the manufacturer. Total RNA (2 µg) was reverse-transcribed to cDNA by MutiScribe Reverse Transcriptase (Thermo Fisher Scientific, REF 4368813). The cDNA (50 ng), primers, and SYBR Green mix (Thermo Fisher Scientific, REFA25742) were mixed and used for qRT-PCR under the condition as follows: Stage 1, 95°C for 10 min; in stage 2, 95°C for 15 sec followed by 60°C for 1 min in a total of 40 cycles. Melt curve: 95°C for 15 sec, 60°C for 1 min and 95°C for 15 sec in a total of 1cycle. The primers used for qRT-PCR were shown in **Supplementary Table 1**.

### Determination of endotoxin levels in purified Exdn-4

Endotoxin levels in purified Exdn-4 from *N. benthamiana* were determined using the Pierce™ Chromogenic Endotoxin Quantitation Kit (Thermo Fisher Scientific, Cat. no. A39552S).

### Activity assay of plant-produced Exdn-4 on glucose-induced insulin secretion from pancreatic β-cell line MIN6m9

The activity of Exdn-4 produced in *N. benthamiana* for insulin secretion was measured using mouse pancreatic β-cell line MIN6m9. Briefly, MIN6m9 cells were cultured in DMEM at a 11 mM glucose, 5 nl/ml 2-mercaptoethanol, 100 units/ml penicillin G, 100 µg/ml streptomycin-sulphat and 10% (v/v) fetal bovine serum (FBS). Once the cells had reached approximately 80% confluence, they were seeded into a 24-well plate at a density of 1.25 × 10^5^ cells per well. In the experiment day, they were pre-incubated in 500 μL of a modified KREBS buffer containing 119 mM NaCl, 4.6 mM KCl, 2.8 mM CaCl_2_, 1 mM MgSO_4_, 0.15 mM Na_2_HPO_4_, 0.4 mM KH_2_PO_4_, 5 mM NaHCO_3_, 0.5 mg/mL BSA and 20 mM HEPES, pH 7.4 with 0.5 mM glucose for 60 min, and were stimulated in 500 μL of the KREBS buffer with 0.5 mM or 5 mM glucose together with varying concentrations of Exdn-4 at 37°C for 20 min. After incubation for 20 min, cell culture supernatants were collected, and the insulin content was measured using a mouse insulin ELISA Kit (ALPCO Diagnostics, Salem, NH, USA) according to the manufacturer’s instructions. The amount of glucose-induced insulin secretion was normalized using total protein from the cells which was determined by BCA assay (ThermoFisher Scientific, Cat no. 23227)

### Statistical analysis

Data are expressed as mean ± SEM. The data were statistically analyzed by Graphpad Prism software version 9.4.1. For the statistical analysis of glucose-induced insulin secretion, we did Shapiro-Wilk test to ensure normal distribution. The test showed that we could reject that our results were not in normal distribution. For equal variances, we did Brown-Forsythe test and it showed, p=0.4775, meaning that variances of each variable were not significantly different. Then, two way ANOVA followed by Turkey’s multiple comparison was performed where alpha level (significance level) was set to 0.05.

## Results

### Exdn-4 containing recombinant proteins are expressed at a high level in *Nicotiana benthamiana*

**Figure 1:**
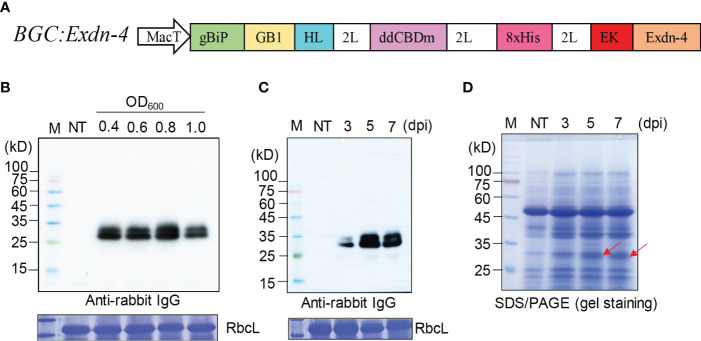
A recombinant gene containing Exdn-4 is expressed at high levels in *N. benthamiana.* **(A)** Schematic presentation of the recombinant construct. BGC : Exdn-4 contains various domains for high-level expression. gBiP, a genomic DNA fragment of Arabidopsis *BiP1* encoding the signal peptide for ER targeting; GB1, B1 domain of streptococcal G protein; HL, a helical wheel-forming linker; 2L, 2 copies of a flexible linker GGGS; ddCBDm, two copies of CBD1 with a mutation of tyrosine 34 to histidine; 8xHis, eight His residues; EK, enterokinase cleavage site; MacT, MacT promoter. **(B, C)** Optimization for the expression of recombinant construct. **(B)** *N. benthamiana* leaves were infiltrated with different *Agrobacterium* concentrations (0.4, 0.6, 0.8, and 1.0 of OD_600_) and leaf tissues were harvested at 5 dpi. **(C)** Plants infiltrated with *Agrobacterium* at 0.8 of OD_600_ were grown for 3, 5, or 7 days after infiltration. Total protein extracts from leaf tissues were separated by SDS/PAGE and analyzed by Western blotting using HRP-conjugated anti-rabbit IgG antibody. M, protein size standard; NT, non-transformed wild-type plants. RbcL stained with CBB, a loading control. **(D)** SDS/PAGE analysis for the expression of BGC : Exdn-4. Total soluble protein extracts (10 μg) were separated by SDS/PAGE and the gel was stained with CBB to detect the protein band of BGC : Exdn-4. Arrows, the expected size of BGC : Exdn-4; Lane M, protein size standard; NT, non-transformed wild-type *N. benthamiana*.

To express Exdn-4 in plants, we designed a recombinant gene containing Exdn-4. Since Exdn-4 is a small polypeptide, it may not be expressed at a high level in the endoplasmic reticulum (ER) and may also be difficult to purify. Thus, we fused to Exdn-4 a few domains that were intended for solubilization of recombinant protein, ER targeting, affinity tagging for purification, and also translational enhancement. First, we utilized the GB1 domain, which is known to increase the expression level of its fusion proteins several-fold (Song et al., 2022). In addition, we intended to accumulate proteins in the ER by targeting recombinant proteins to the ER using the signal peptide of Arabidopsis BiP1, including seven amino acids after the signal peptide cleavage site to favor proper peptide removal. However, we decided not to use the ER retention motif because we aimed to produce Exdn-4 without any extra amino acid residues. As an affinity tag for protein purification, we included two domains, a double CBD1 domain with a mutation of tyrosine 31 to histidine (ddCBDm) and 8 histidine residues (8xHis). CBD domains are known to increase the solubility of the fusion proteins and also can be used as a purification tag using microcrystalline cellulose (MCC) beads. 8xHis can also be used as an epitope tag for Western blot analysis and as an affinity tag for protein purification using Ni^2+^-NTA resin. Finally, an enterokinase site (DDDDK) and the Exdn-4 domain were fused to the C-terminus of ddCBDm to give BiP : GB1:ddCBDm:8xHis : EK:Exendin-4 (*BGC : Exdn-4* for short) (**Figure 1A**). The recombinant gene was under the control of MacT promoter (Song et al., 2022). It also contained the gene silencing suppressor *P38* of turnip crinkle virus coat protein, so there was no need to provide *P38* in a separate vector. It has been shown to enhance the recombinant protein expression in *N. benthamiana* (Thomas et al., 2003).

We examined the expression of the recombinant gene, *BGC : Exdn-4*, in *N. benthamiana* by *Agrobacterium*-mediated infiltration (Zhang et al., 2020). First, we tested the optimal *Agrobacterium* concentration for infiltration using various concentrations of *Agrobacterium* suspension: 0.4, 0.6, 0.8, and 1.0 of OD_600_. Total soluble protein extracts from leaf tissues harvested at 5 dpi were analyzed by Western blotting using the secondary antibody, anti-rabbit IgG antibody, which detected the GB1 domain of BGC : Exdn-4 (Song et al., 2022). The expression of BGC : Exdn-4 increased along with *Agrobacterium* concentration up to 0.8 of OD_600_ and then decreased to a lower level at 1.0 of OD_600_ (**Figure 1B**). Then, we examined the expression level according to the growth period after infiltration. We harvested plant leaf tissues at three different time points: 3, 5 and 7 dpi. Again, total soluble protein extracts were analyzed by Western blotting using the secondary antibody, HRP-conjugated anti-rabbit IgG antibody. The expression level of BGC : Exdn-4 was highest at 5 dpi (**Figure 1C**; **Supplementary Figure 1**). Unlike previous study (Buyel and Fischer, 2012; Gengenbach et al., 2019), the expression level increased from 3 dpi to 5 dpi but then slightly decreased at 7 dpi. To examine whether BGC : Exdn-4 can be detected by CBB staining of an SDS/PAGE gel, 10 μg of total soluble proteins were separated by SDS/PAGE and the gel was stained with CBB (**Figure 1D**). The recombinant protein BGC : Exdn-4 was detected on the CBB-stained gel, confirming that BGC : Exdn-4 is expressed at a high level. We compared the expression level of BGC : Exdn-4 with the same construct with an ER retention motif HDEL at the C-terminus. In general, the ER retention motif is added to the C-terminus of recombinant proteins to increase the expression level because the ER retention motif usually induces the accumulation of recombinant proteins in the ER at high levels (Song et al., 2022). Thus, we generated a construct *BGC : Exdn-4:HDEL* and expressed it in *N. benthamiana*. The expression levels of both constructs were nearly the same (**Supplementary Figure 2**), indicating that in the case of BGC : Exdn-4, the ER retention motif does not contribute to the protein yield in plants. To examine the transcript level of these two constructs, we performed qRT-PCR and found that the transcript levels of both *BGC : Exdn-4* and *BGC : Exdn-4*:*HDEL* were nearly the same (**Supplementary Figure 3**).

**Figure 2:**
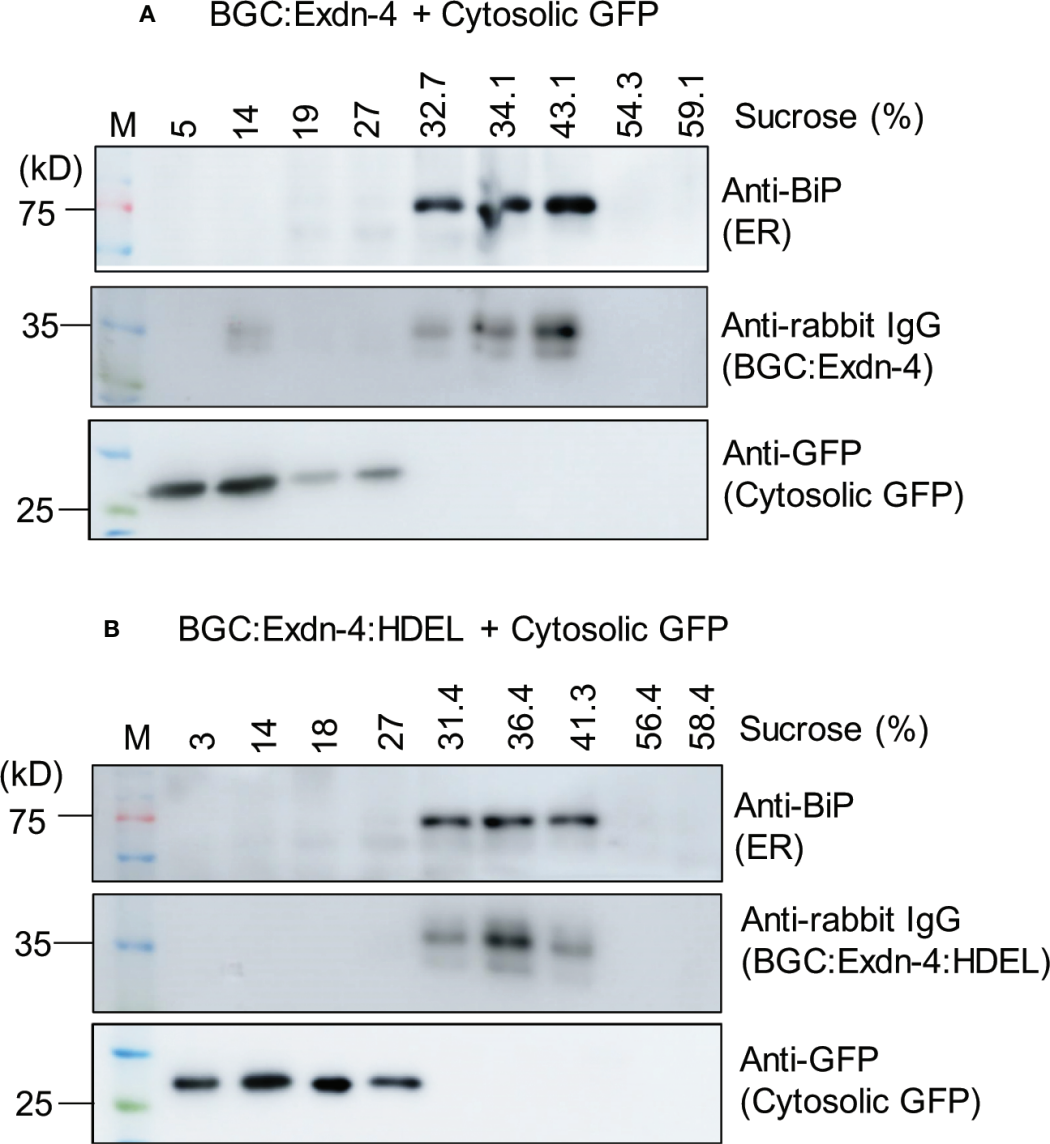
Both BGC : Exdn-4 and BGC : Exdn-4:HDEL expressed in *N. benthamiana* localize to the ER. **(A, B)** Subcellular fractionation and western blot analysis of BGC : Exdn-4 and BGC : Exdn-4:HDEL. Total protein extracts from leaf tissues of *N. benthamiana* expressing *BGC : Exdn-4* and cytosolic *GFP* **(A)**, or *BGC : Exdn-4:HDEL* and cytosolic *GFP* **(B)** were fractionated using a linear sucrose gradient from 10 to 60% sucrose. These fractions were separated by SDS/PAGE and analyzed by western blotting using anti-BiP, anti-rabbit IgG and anti-GFP antibodies. ER proteins were detected in the fractions containing 32.7 to 43.1% sucrose **(A)** or 31.4 to 41.3% sucrose **(B)** whereas the cytosolic GFP was detected in the fractions 5 to 27% sucrose **(A)** or 3 to 27% **(B)**. These results indicate that both BGC : Exdn-4 and BGC : Exdn-4:HDEL localize to the ER.

Next, we examined the localization of both BGC : Exdn-4 and BGC : Exdn-4:HDEL. BGC : Exdn-4:HDEL should be localized to the ER. In contrast, it was not clear whether BGC : Exdn-4 without the ER retention motif also localizes to the ER. Homogenates from infiltrated leaf tissues were fractionated on a linear sucrose gradient using 10 to 60% solution, to separate the different subcellular compartments, and proteins in the fractions were analyzed by western blotting. Both BGC : Exdn-4 and BGC : Exdn-4:HDEL comigrated with endogenous BiP at the fractions from 31 to 43% sucrose whereas coexpressed cytosolic GFP was detected at the top fractions from 3 to 27% sucrose. These results indicate that BGC : Exdn-4 localizes to the ER, despite the absence of the ER retention motif (**Figure 2**).

### BGC : Exdn-4 recombinant proteins are readily purified using either Ni^2+^-NTA resin or microcrystalline cellulose beads

**Figure 3:**
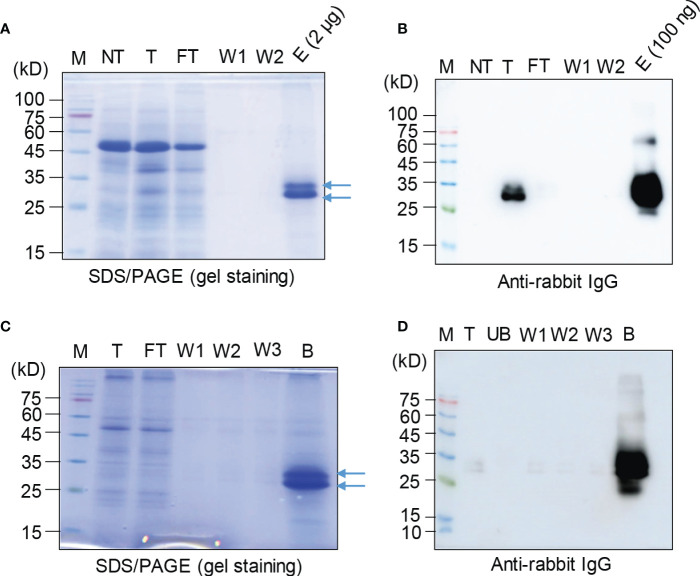
Purification of BGC : Exdn-4 by Ni^2+^-NTA resin and microcrystalline cellulose resin. **(A, B)** Ni^2+^-NTA-based purification of BGC : Exdn-4. Leaf tissues harvested at 5 dpi were homogenized in extraction buffer at a 1:6 ratio (w/v). The total protein extracts were loaded into a column containing Ni^2+^-NTA resin at 4°C, allowed to pass through the column due to gravity, and washed five times using washing buffer. Finally, proteins were eluted using 400 mM imidazole. The indicated fractions from purification were separated by SDS/PAGE and stained by CBB **(A)** or analyzed by Western blotting using HRP-conjugated anti-rabbit IgG antibody **(B)**. M, protein size standard; NT, non-transformed wild-type plants; T, total soluble proteins; FT, flow-through fraction; W1 and W2, the first and second wash-off fractions; E, eluted fraction. Arrows, BGC : Exdn-4. **(C, D)** MCC bead-based purification of BGC : Exdn-4. Total protein extracts from leaf tissues harvested at 5 dpi were mixed with MCC beads followed by washing five times with distilled water. During purification, total protein extracts (T); unbound fraction (UB); and the first, second, and third wash-off fractions (W1, W2, and W3, respectively) were saved for analysis. The B fraction was prepared by releasing proteins from MCC beads in SDS sample buffer by boiling for 10 min. These samples were separated and analyzed by SDS/PAGE and Western blotting **(D)**.

Next, we asked whether the recombinant protein BGC : Exdn-4 can be easily purified from total soluble protein extracts. We used two different types of affinity resins, Ni^2+^-NTA and MCC beads. Total soluble proteins prepared from leaf tissues were subjected to purification as described in the Method section. First, we examined the purification using Ni^2+^-NTA resin. The purification steps were monitored by SDS/PAGE followed by CBB staining or Western blotting using the secondary antibody, anti-rabbit IgG antibody. The total soluble protein extracts, flow-through fraction, wash-off fractions, and eluted proteins were separated by SDS/PAGE and stained by CBB. The CBB-stained gel image showed that the majority of host proteins were detected in the flow-through fraction and almost no protein was detected in wash-off fractions. In the eluents, two closely migrating bands were detected at the position between 25 and 35 kD (**Figure 3A**). This is the expected size based on the calculated molecular mass. However, the bottom band was stronger than the upper band, raising the possibility that a small portion of BGC : Exdn-4 is N-glycosylated. Next, we examined the identity of these proteins using HRP-conjugated anti-rabbit IgG antibody, (**Figure 3B**). The total extracts produced two bands, as in the case of CBB-stained gel. In addition, the band at 27 kD was strongly detected by the anti-rabbit IgG, confirming that the 27-kD band represents BGC : Exdn-4. The flow-through and wash-off fractions did not show any protein bands, indicating that BGC : Exdn-4 binds to Ni^2+^-NTA tightly. We quantified the purified protein by comparing the amount of purified proteins with the known amounts of human serum albumin (HSA) on a SDS/PAGE gel after CBB staining (**Supplementary Figure 4**). Quantification indicates that the yield of BGC : Exdn-4 *N. benthamiana* was approximately 45 µg/g fresh biomass.

Next, we used MCC beads to purify BGC : Exdn-4 recombinant proteins. MCC beads are inexpensive and natural resource that has been used for the purification of recombinant proteins from total protein extracts from leaf tissues (Islam et al., 2019). A previous study showed that MCC beads have a binding capacity of 2 μg/mg of proteins, with CBM3 as an MCC binding domain (Islam et al., 2019). First, we assessed the binding capacity of MCC beads for ddCBDm as the affinity tag using the total protein extracts. Varying amounts of total soluble protein extracts were incubated with 10 mg of MCC beads. The mixture was separated into the supernatant that contained the unbound proteins and the pellet that contained the MCC beads bound to BGC : Exdn-4. These fractions were analyzed by Western blotting using anti-rabbit IgG antibody. BGC : Exdn-4 was not detected in the supernatant until 600 μL of total soluble protein extracts but detected at both 800 and 1000 μL (**Supplementary Figure 5**), indicating that BGC : Exdn-4 in 600 μL of total soluble protein extracts is the saturation point for 10 mg of MCC beads under the conditions we used to prepare the total soluble protein extracts.

Based on this binding capacity of MCC beads for BGC : Exdn-4, we purified BGC : Exdn-4 using MCC beads. Total protein extracts were prepared from *N. benthamiana* leaf tissues harvested at 5 dpi and mixed with MCC beads. The MCC beads were washed extensively to remove loosely bound non-specific proteins. To evaluate the purification process of BGC : Exdn-4 recombinant proteins, all the fractions, including the total, flow-through, wash-off solutions, and eluted proteins from MCC beads by boiling, were separated by SDS/PAGE and stained by CBB or analyzed by Western blotting using anti-rabbit IgG antibody. The CBB-stained gel showed that the majority of proteins were detected in the flow-through fraction and minor bands in the wash-off fraction. The eluents contained the two bands of BGC : Exdn-4 at the 27- and 30-kD positions as in the case of MCC purification (**Figure 3C**). Western blot analysis confirmed that the band around 27 kD represents BGC : Exdn-4 (**Figure 3D**). Moreover, anti-rabbit IgG did not detect any bands in the unbound and washing fractions, indicating that BGC : Exdn-4 strongly binds to MCC beads. Purified proteins were quantified to estimate the purification efficiency using MCC beads. When we compared the amount of purified proteins, it appeared that MCC beads were slightly better than Ni^2+^-NTA resin (**Supplementary Table 2**). We also quantify the total soluble Exdn-4 protein expression in *N. benthamiana* (**Supplementary Figure 6**).

**Figure 4:**
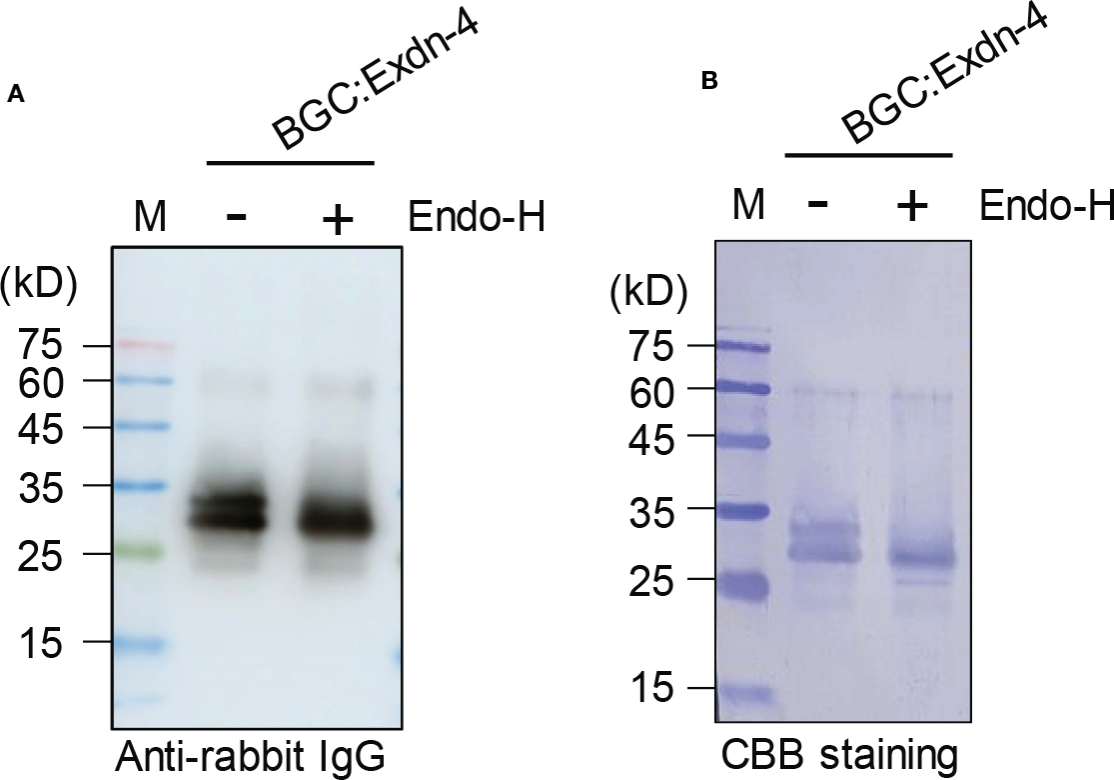
A minor proportion of BGC : Exdn-4 is N-glycosylated in *N. benthamiana.* Purified BGC : Exdn-4 (2 μg) was denatured by boiling and then incubated with 0.5 units of Endo-H at 37°C for 2 h. Endo-H-treated (+) and untreated (-) BGC : Exdn-4 were separated by SDS/PAGE followed by Western blot analysis **(A)** or CBB staining **(B)**. M, protein size standard.

BGC : Exdn-4 produced two bands at the 25- and 30-kD positions. We postulated that the upper band is the N-glycosylated form. To test this idea, we treated the purified BGC : Exdn-4 with endo-H and analyzed it by Western blotting using anti-rabbit IgG antibody. The upper band disappeared upon endo-H treatment (**Figure 4**), indicating that the upper band is N-glycosylated. The N-terminal BGC region contained an N-glycosylation site (**Supplementary Figure 7**).

Next, we examined the endotoxin level of the purified recombinant Exdn-4. It has been proposed that plant-produced proteins are safer regarding contamination of endotoxins. However, we used *Agrobacterium*-mediated infiltration to deliver the target gene. *Agrobacterium* is a Gram-negative bacterium that contains endotoxin in the cell wall. Thus, we measured the amount of endotoxin in the purified protein using the LAL test. The amount of endotoxin was approximately 0.04 EU/μg of BGC : Exdn-4 (**Supplementary Figure 8**), confirming very low levels of endotoxin contamination in the purified protein.

### Exdn-4 without any extra amino acid residues is produced by enterokinase-mediated proteolytic cleavage

**Figure 5:**
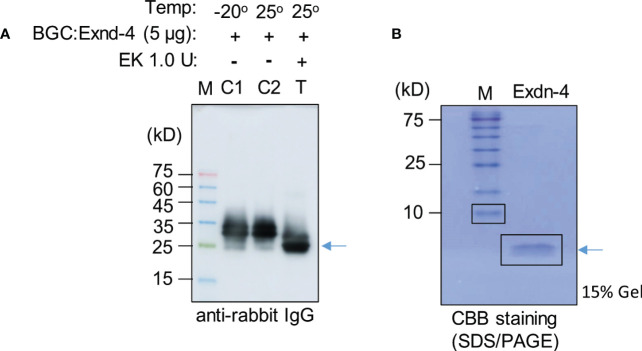
Exdn-4 is produced without any extra domains by cleavage of BGC : Exdn-4 with enterokinase. **(A)** Enterokinase cleavage of BGC : Exdn-4. Purified BGC : Exdn-4 bound onto MCC beads (50 µL/5 µg proteins) beads was treated with (T) or without (C2) 1.0 U of Enterokinase at 25°C for 12 h. After incubation, proteins bound to MCC beads were released in the SDS/PAGE sample buffer by boiling and analyzed by Western blotting using HRP-conjugated anti-rabbit IgG antibody. As a control, untreated protein (C1) was loaded. Arrow, BGC without Exdn-4. **(B)** Analysis of Exdn-4 released from BGC : Exdn-4. Exdn-4 released in the supernatant during the enterokinase treatment was collected and analyzed in by 15% SDS/PAGE gel followed by CBB staining. Arrow, Exdn-4.

We examined whether Exdn-4 without any extra domains can be produced from BGC : Exdn-4 (**Supplementary Figure 9**). Purified BGC : Exdn-4 that was still bound to MCC beads was treated with or without enterokinase at 25°C for 12 h. To examine whether Exdn-4 is released from BGC : Exdn-4 by the enterokinase treatment, the buffer solution that would contain the released Exdn-4 was collected from the digestion mixture and also proteins bound to MCC beads were released by boiling. These protein samples were analyzed by Western blotting using HRP-conjugated anti-rabbit IgG antibody. As a control, we included proteins stored at -20°C. The enterokinase-treated sample showed a protein band smaller than the original protein (**Figure 5A**), indicating that the C-terminus Exdn-4 is released from BGC : Exdn-4. In addition, the full length was not detected upon EK treatment, an indication of efficient cleavage by enterokinase. Moreover, the N-terminal domain was still bound to the MCC beads. In contrast, there was no difference in size and amount of BGC : Exdn-4 without enterokinase treatment, indicating that the protein is stable at 25°C during 12 h incubation. Next, we analyzed the supernatant to detect the released Exdn-4 using 15% SDS/PAGE followed by CBB staining. A protein band was detected at the position below 10 kD, the expected size of Exendin-4 (**Figure 5B**), confirming that Exdn-4 can be produced from the BGC : Exdn-4.

### Plant-produced Exdn-4 potentiates glucose-induced insulin secretion

**Figure 6:**
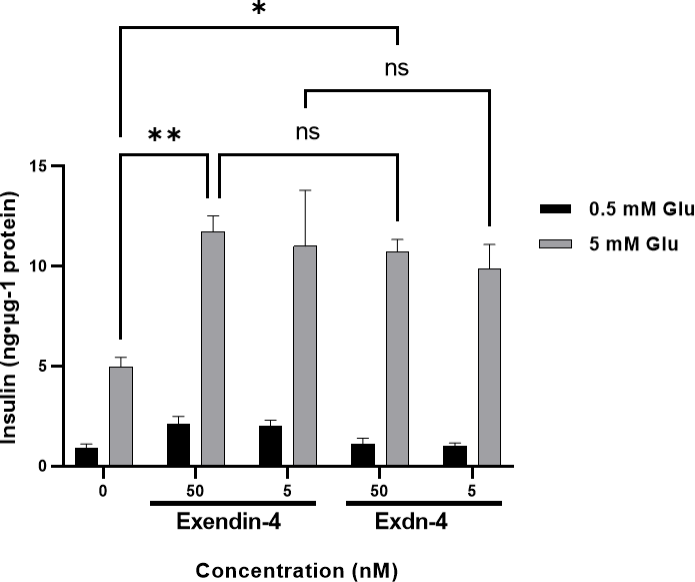
Plant-produced Exdn-4 induces glucose-dependent insulin secretion from MIN6m9 pancreatic β-cells. MIN6m9 cells were treated with plant-produced Exdn-4 or chemically synthesized Exendin-4 at varying concentrations in the presence of 0.5 mM or 5 mM glucose. Supernatants from treated cells were collected, and the insulin concentration was measured by mouse insulin ELISA. The assay was performed in triplicate and repeated three times (n = 3). Data were expressed as means ± SEM. Statistical analysis was performed using Two-way analysis of variance (ANOVA), followed by Tukey’s multiple comparison analysis with a P value less than 0.05 (p < 0.05) considered as statistical significance.*: p < 0.05, **: p < 0.01 and ns: not significant.

A single dose of Exendin-4 is sufficient to normalize the blood glucose level in diabetic mice. We examined whether our plant-produced Exdn-4 is biologically active under an *in-vitro* cell culture model. Exendin-4 is known to stimulate the release of insulin from pancreatic β-cells in a glucose-dependent fashion (Holst and Gromada, 2004). The pancreatic β-cell line MIN6m9 is used to determine the glucose-induced insulin secretion by Exendin-4 (Cheng et al., 2012). We applied varying concentrations (5, 50 nM) of plant-produced Exdn-4 to MIN6m9 cells in the presence and absence of glucose, and the amount of insulin secreted by the cells was measured by ELISA. As a positive control, we used chemically synthesized Exendin-4. As expected, in the presence of 5 mM glucose, chemically synthesized Exendin-4 potentiated glucose-induced insulin secretion from MIN6m9 cells with statistical significance whereas the basal insulin level remained unchanged in the presence of 0.5 mM glucose. Similarly, plant-produced Exdn-4 also significantly potentiated glucose-induced insulin secretion from MIN6m9 cells, indicating that the plant-produced Exdn-4 is active (**Figure 6**). Moreover, statistical analysis showed that the activity of plant-produced Exdn-4 was not statistically different from one of chemically synthesized Exendin-4.

## Discussion

In this study, we developed a new strategy for the production of recombinant Exdn-4 *in N. benthamiana.* Our main focus was to establish a method to achieve high-level production of active proteins and easy purification with no contamination of endotoxins. In the design of the construct for the production of recombinant Exdn-4, we incorporated two important domains, GB1 and ddCBDm. GB1 was shown to increase the expression level as well as the stability of the fusion protein in plants (Song et al., 2022). ddCBDm was used as an affinity tag for protein purification using MCC beads and is also known to provide solubility of the fusion proteins (Wan et al., 2011; Wang and Hong, 2014). ddCBDm is a fusion of two CBD1 domains designed for strong binding to MCC beads. CBD alone was shown to bind strongly to MCC beads. Thus, we expected that the double CBD domain should have stronger to MCC beads.

Exdn-4 is a small peptide with only 39 amino acid residues. Thus, it is rather small to be produced as a recombinant protein in plant cells. Therefore, we generated a fusion protein construct consisting of GB1, ddCBDm, 8xHis, and Exdn-4. In addition, the recombinant protein was induced to be localized to the ER using the BiP signal peptide. In plants, the ER is thought to be the best place for the accumulation of recombinant proteins at high levels because it provides a suitable environment for correct folding and post-translational modification if necessary and is known to have the least risk of proteolytic degradation (Schouten et al., 1996). The addition of an ER retention motif to the C-terminus could further increase the accumulation of targeting proteins in the ER (Gomord et al., 2002; Wilbers et al., 2016). However, it has been shown that the addition of extra amino acid residues to the C-terminus of Exdn-4 interferes with biological activity (Wakefield et al., 1991). Thus, we excluded the ER retention motif from the construct. Despite the absence of the ER retention motif, we obtained the expression of Exdn-4 recombinant proteins at high levels. Recombinant protein of BGC : Exdn-4 was detectable in the CBB-stained gel when 10 μg of total soluble proteins were separated by SDS/PAGE. This expression level was achieved without adding the ER retention motif in the ER. Moreover, in the case of BGC : Exdn-4, the addition of the ER retention motif did not further increase the expression level. In fact, BGC : Exdn-4 without the HDEL motif was also localized to the ER, which could be the reason why addition of the HDEL motif did not increase the expression level. In plants, RNA or DNA viral vectors are used to achieve a high level of expression of recombinant proteins. These vectors are known to express as much as a few milligrams of recombinant proteins per gram of fresh biomass. Another approach is to express recombinant proteins in the chloroplast after the integration of the recombinant gene in the chloroplast genome. However, in general, using an ordinary binary vector such as pCAMBIA1300, the recombinant protein band were not detectable by the CBB staining (Khan et al., 2022). Thus, our recombinant gene, *BGC : Exdn-4*, used for the production of Exdn-4 is considered to be expressed at a high level. After purification of BGC : Exdn-4, we estimated the yield of purified proteins to be ~50 µg/g fresh biomass.

We developed a highly efficient method for the purification of plant-produced recombinant proteins using MCC beads. There are many affinity tag-based purification methods. However, most of them are expensive. In fact, the price of recombinant proteins largely depends on the purification cost; the purification steps accounting for 70–80% of the production costs (Raven et al., 2014). We used the double CBD1 domain as the affinity tag for purification of Exdn-4 recombinant proteins. Both CBM3 and CBD strongly bind to MCC beads (Wan et al., 2011; Wang and Hong, 2014) and are considered as promising affinity tags. MCC beads are inexpensive natural materials. Thus, an advantage of using MCC beads as an affinity resin is the low cost. Using MCC beads, we were able to purify the recombinant proteins in a single step. However, one potential disadvantage is that the proteins bound to MCC beads cannot be easily released from the MCC beads if one wants to elute them. In this study, we aimed to produce Exdn-4 without any extra residues. Thus, we designed the recombinant construct of Exdn-4 in such a way that the N-terminus domain should be removed by enterokinase-mediated proteolytic cleavage. In fact, Exdn-4 was easily released from the recombinant proteins bound to MCC beads by enterokinase-mediated cleavage, which suggested that the on-bound cleavage of Exdn-4 by enterokinase occurred efficiently. We also used Ni^2+^-NTA resin to purify BGC : Exdn-4. The recombinant proteins were easily purified in a single step as well at a high purity. In the case of Ni^2+^-NTA resin, one can reuse it multiple times to reduce the cost of the resin.

An important question regarding the production of recombinant proteins in a heterologous system is whether the target protein is active in the original organism. In particular, plants belong to a different kingdom than animal cells. Thus, it is essential to test the activity of proteins produced in plants. Indeed, plant-produced Exdn-4 has activity comparable to chemically synthesized Exendin-4 when we tested it based on glucose-dependent insulin secretion in MIN6m9 cells. In this study, we focused on the production of Exdn-4 without any extra residues. However, it is possible that plant-produced Exdn-4 recombinant proteins can also be used for oral administration with or without purification. This will add more benefits to using plants as a host for the production of Exdn-4. This will be a direction of future study.

In this study, we developed an Exdn-4 production system using plants as a production host. From this study to real commercialization, one crucial point is how to translate the protocol we developed in the laboratory at a small scale to a commercial scale. Many steps such as centrifugation and grinding of tissues that were used in the laboratory may not be applicable to the commercial scale. These aspects should be studied further in the future for real commercialization.

## Data availability statement

The original contributions presented in the study are included in the article/**Supplementary Material**. Further inquiries can be directed to the corresponding author.

## Author contributions

SA and IH conceived the project. SA and IH designed the research. SA performed most of the experiments. SJA and JK performed the activity assay of Exdn-4. P-OB was involved in conceiving the idea for this study. JHK aided primer design and MR performed agroinfiltration with SA. SA and IH interpreted the results and wrote the manuscript. All authors contributed to the article and approved the submitted version.
