# Supplementary material for: Production of active Exendin-4 in Nicotiana benthamiana and its application in treatment of type-2 diabetics
Source: Front Plant Sci. 2022 Dec 22;13:1062658. doi: 10.3389/fpls.2022.1062658 (PMC9812950; doi:10.3389/fpls.2022.1062658)
Supplement: Supplementary file 1 [file DataSheet_1.docx]

**Production of active Exendin-4 in *Nicotiana benthamiana* and its application in treatment of type-2 diabetics**

**Shammi Akter^1^, Shajia Afrin^2^, Jaeyoon Kim^2^, Joohyun Kang^1^, Md Abdur Razzak^1^, Per-Olof Berggren^3^, Inhwan Hwang^1^**

**^1^Department of Life Sciences, Pohang University of Science and Technology,**

**Pohang, South Korea**

**^2^BioN Inc., Pohang, South Korea**

**^3^Department of Molecular Medicine and Surgery, The Rolf Luft Research Center for Diabetes and Endocrinology, Karolinska Institutet, Stockholm, Sweden**

^*^Corresponding author: To whom correspondence should be addressed

E-mail: [ihhwang@postech.ac.kr](mailto:ihhwang@postech.ac.kr)

Tel: 82-54-279-2128

Fax: 82-54-279-8159

ORCID ID: 0000-0002-1388-1367 (I.H.)

**Supplementary Figures**


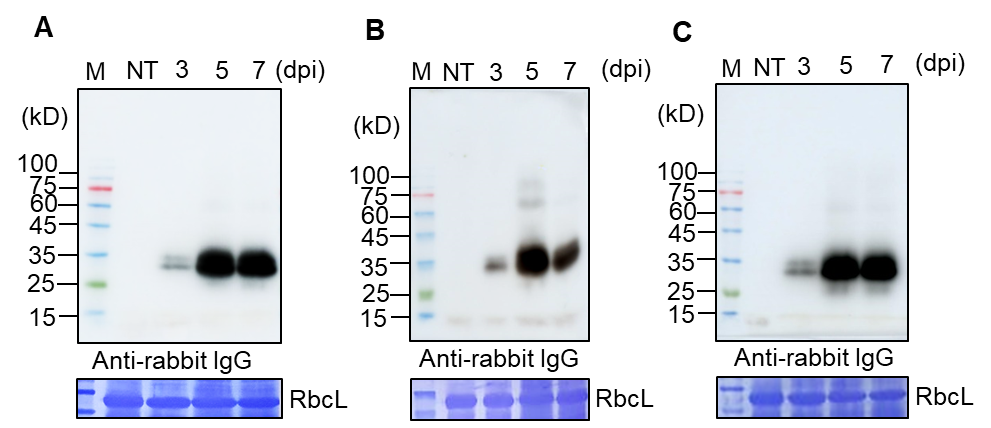


**Supplementary Figure 1. Expression of BGC:Exdn-4 at various time points after infiltration.**

Agro-infiltrated leaf tissues were collected 3, 5 and 7 dpi and used to prepare total protein extracts. We extracts total proteins of 3, 5 and 7 dpi and loaded 10 µg of total protein in each lane of SDS-PAGE and analyzed by western blotting using anti-rabbit IgG antibody. (**A**), (**B**) and (**C**) are 3 independent experiment (biological replicates) to get the reproducibility.


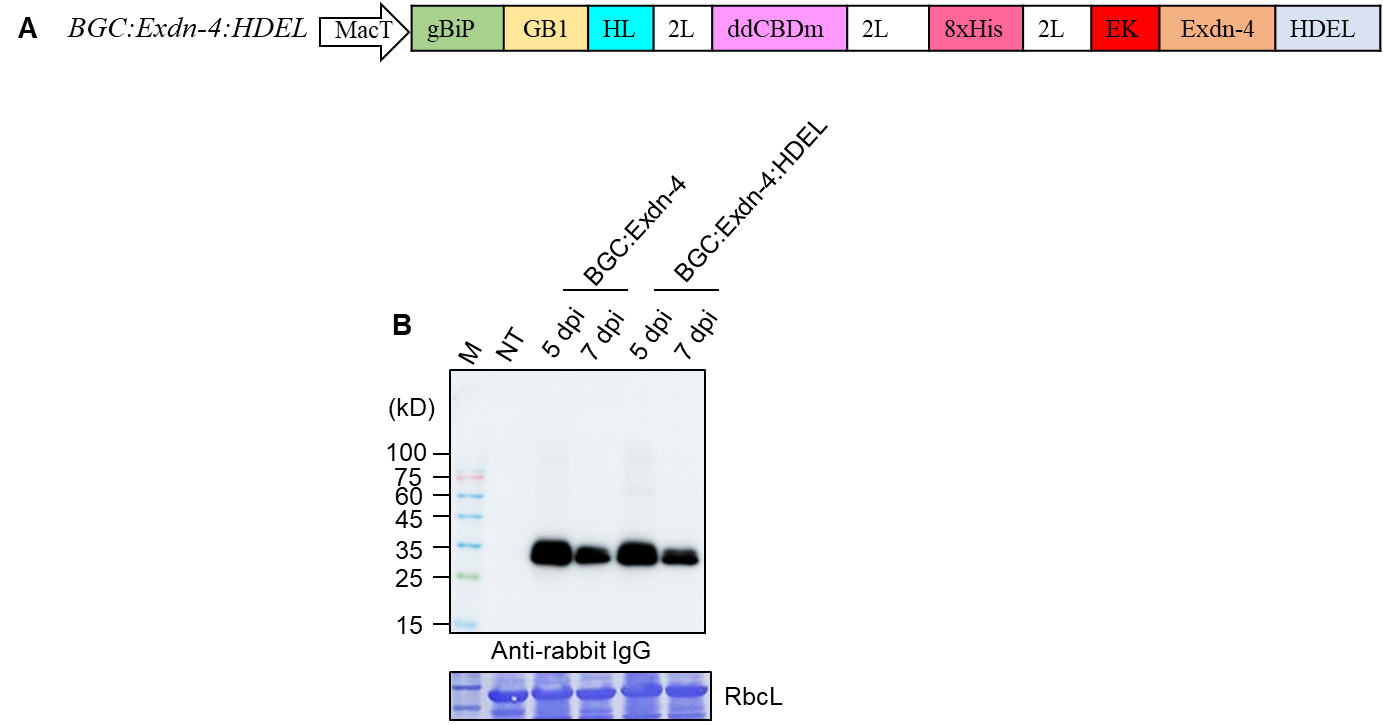


**Supplementary Figure 2. The ER retention motif HDEL at the C-terminus of Exdn-4 does not increase the expression level.**

(**A**) Construction of HDEL-containing *BGC:Exdn-4* construct. The ER retention motif HDEL was added to the C-terminus of 2L:EK:Exdn-4 by PCR using primers XmaI-2L-EK-F and XhoI-HDEL-R (Supplemental Table 1). Amplified PCR product was digested with *XmaI* and *XhoI* restriction endonucleases and ligated into pTEX1::B:G:ddCBDm:Exdn-4, digested with *Xm****aI* and *Xho1* restriction endonucleases to give** pTEX1::B:G:ddCBDm:Exdn-4:HDEL (*BGC:Exdn-4:HDEL*).

(**B**) Expression of *BGC:Exdn-4 and BGC:Exdn-4:HDEL*. Both constructs were introduced into leaf tissues of *N. benthamiana* for expression. Total soluble protein extracts (10 μg) from leaf tissues harvested at 5 and 7 dpi were analyzed by Western blotting using HRP-conjugated anti-rabbit IgG antibody. Lane M, protein size standard; NT, non-transformed wild-type *N. benthamiana*.


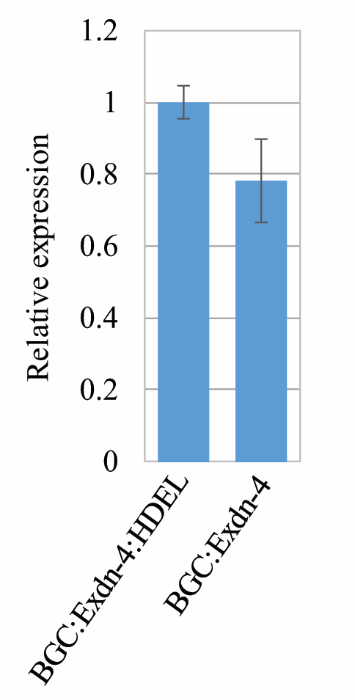


**Supplementary Figure 3. Transcript level analysis of BGC:Exdn-4 and BGC:Exdn-4:HDEL.**

*BGC:Exdn-4* and *BGC:Exdn-4:HDEL* were transiently expressed in *Nicotiana benthamiana* via *Agrobacterium*-mediated infiltration. Total RNA was prepared from leaf tissues at 5 dpi and used for qRT-PCR. Actin (ACT3) of *Nicotiana benthamiana* was used as an internal control for qRT-PCR. As a control for Agrobacterium infiltration, *P38* carried by the same expression vector was used. *BGC:Exdn-4* and *BGC:Exdn-4:HDEL* gene expression was normalized to internal control Actin by 2^-ΔΔCT^ method (Livak et al., 2001). The expression level of *BGC:Exdn-4* was represented as a relative value to that of *BGC:Exdn-4:HDEL*. Results are the mean SE (n = 3). Error bars indicate standard errors (± SE) of three independent experiments. Statistical analysis was performed using an unpaired t-test (GraphPad Prism 9) where a P value less than 0.05 (P ≤ 0.05) was considered to be statistical significance. The results indicated that there was no significant difference in the expression level between the two samples.

**
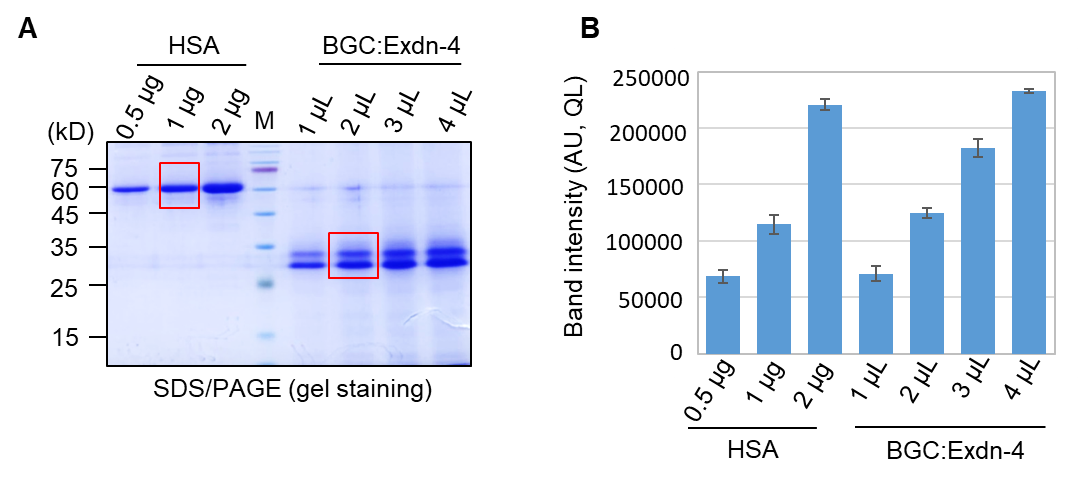
**

**Supplementary Figure 4. Quantitative analysis of purified BGC:Exdn-4.**

**(A)** Quantitative analysis of purified BGC:Exdn-4. To quantify the protein expression in the plant, different amounts (1 to 4 μL) of purified BGC:Exdn-4 were separated by SDS/PAGE. We included a standard protein, human serum albumin (HSA), to compare the amount of BGC:Exdn-4. We observed that 2 μL of BGC:Exdn-4 are equivalent to a band intensity of 1 µg HSA. The signal intensity of the protein bands was measured using Multi Gauge V2.2 densitometric software (Fujifilm).

**(B)** The quantity of BGC:Exdn-4 was represented in arbitrary units (QL, in pixels) at a log-10 scale (Log10). According to the band intensity and total volume of elution, the amount of purified proteins from 40 g of leaf was 1.820 mg. Quantification indicates that the yield of BGC:Exdn-4 *N. benthamiana* was approximately 45 µg/g fresh biomass.

**
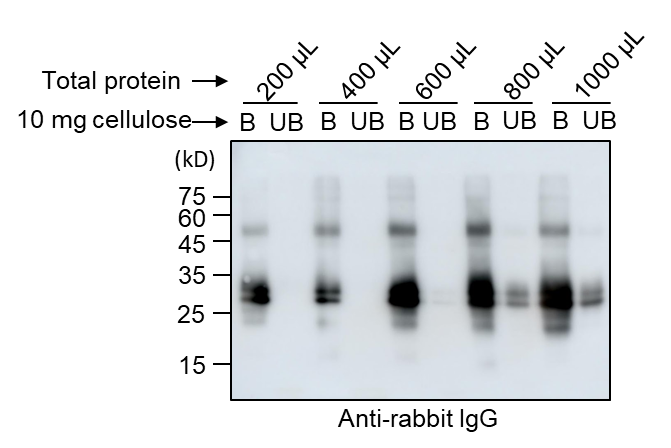
**

**Supplementary Figure 5. Assessment of binding capacity of MCC beads for BGC:Exdn-4.**

To assess the binding capacity of MCC beads to BGC:Exdn-4, total protein extracts were prepared in a volume of 4 mL of extraction buffer from leaf tissues (2 g fresh biomass) harvested at 5 dpi. Varying volumes (200–1000 µL) of total protein extracts were incubated with 10 mg of MCC beads. After binding, MCC beads and the supernatant (unbound fraction) were collected separately. MCC beads harboring bound proteins were washed four times with 40 mM Tris-HCl, pH 7.5. Proteins bound to beads (B fraction) were released in 50 µL of protein sample buffer by boiling. Both bound and unbound fractions were separated by 12% SDS/PAGE and analyzed by Western blotting using HRP-conjugated anti-rabbit IgG antibody.


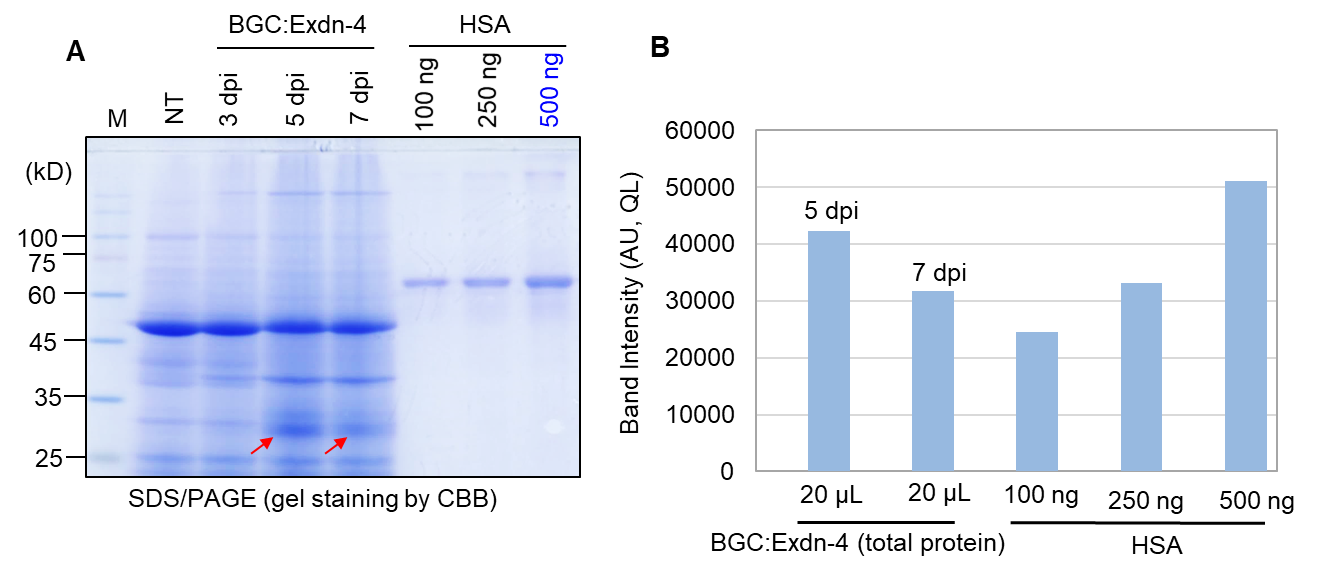


**Supplementary Figure 6. Quantification of the BGC:Exdn-4 protein level in *N. benthamiana.***

**(A)** Agro-infiltrated of *BGC:Exdn-4* leaf tissues were collected at 3 dpi, 5 dpi, 7 dpi. We added 600 μL of extraction buffer to 250 mg powder of 3, 5 and 7 dpi leaf tissues. To calculate the percentage of BGC:Exdn-4 in the total proteins, we loaded 20 μL (20.4 μg) of total protein in an SDS/PAGE gel (protein concentration measure by Bradford assay). We included human serum albumin (HSA) to compare the percentage of BGC:Exdn-4 in total soluble proteins. We found that BGC:Exdn-4 in 20 μg of total soluble proteins was equivalent to a band intensity of 0.450 µg of HSA. **(B)** The intensity of the protein bands was measured using Multi Gauge V2.2 densitometric software (Fujifilm). From this calculation, we found that BGC:Exdn-4 was 2.2% of total soluble proteins in *N. benthamiana.*

**
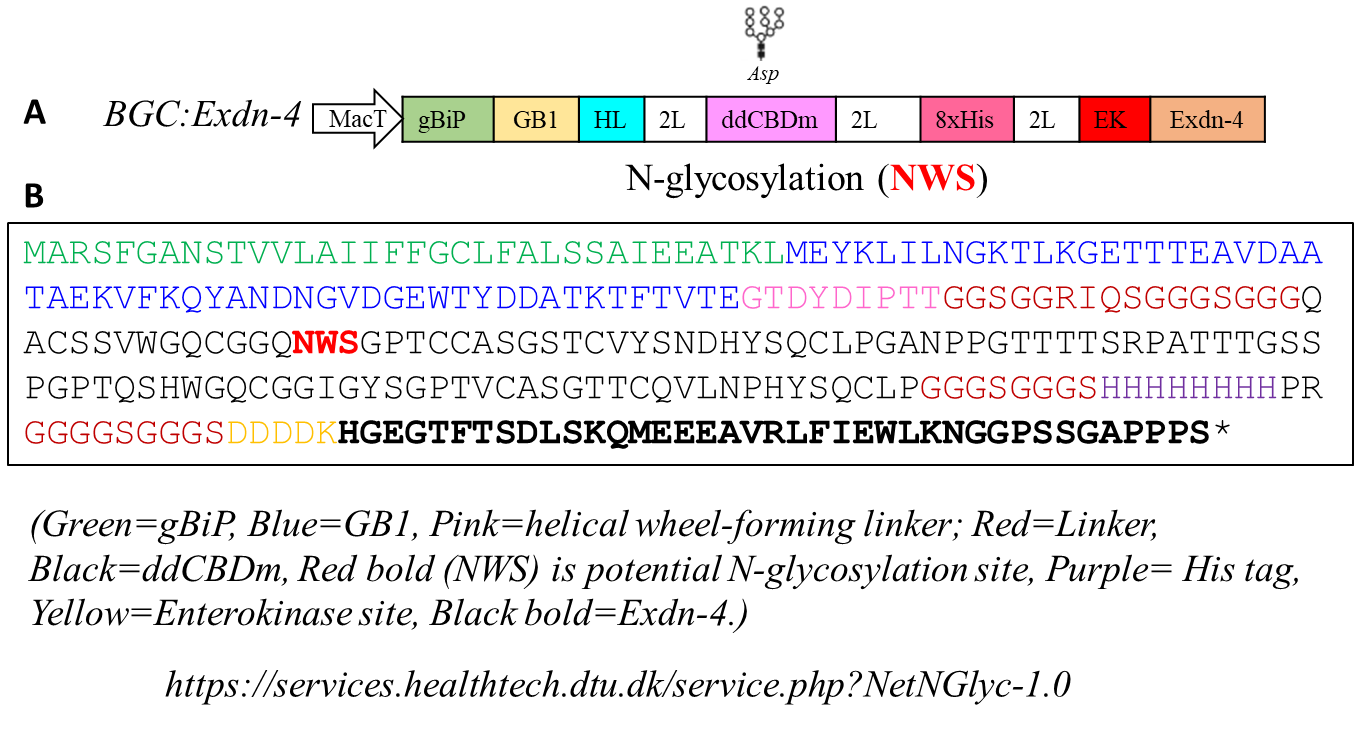
**

**Supplementary Figure 7. The amino acid sequence of the chimeric construct.**

(A) The vector map showing the position of potential N-glycosylation site. (B) The amino acid sequence of the chimeric construct. Various domains in the construct were indicated in different colors. Green, are the first 34 amino acids of BiP1 which named as gBiP; Blue, GB1; Pink, a helical wheel-forming linker; Red, Linker; Black, ddCBDm; Red bold (NWS), N-glycosylation site; Purple, His tag; Yellow, Enterokinase site; Black bold, Exdn-4.

**
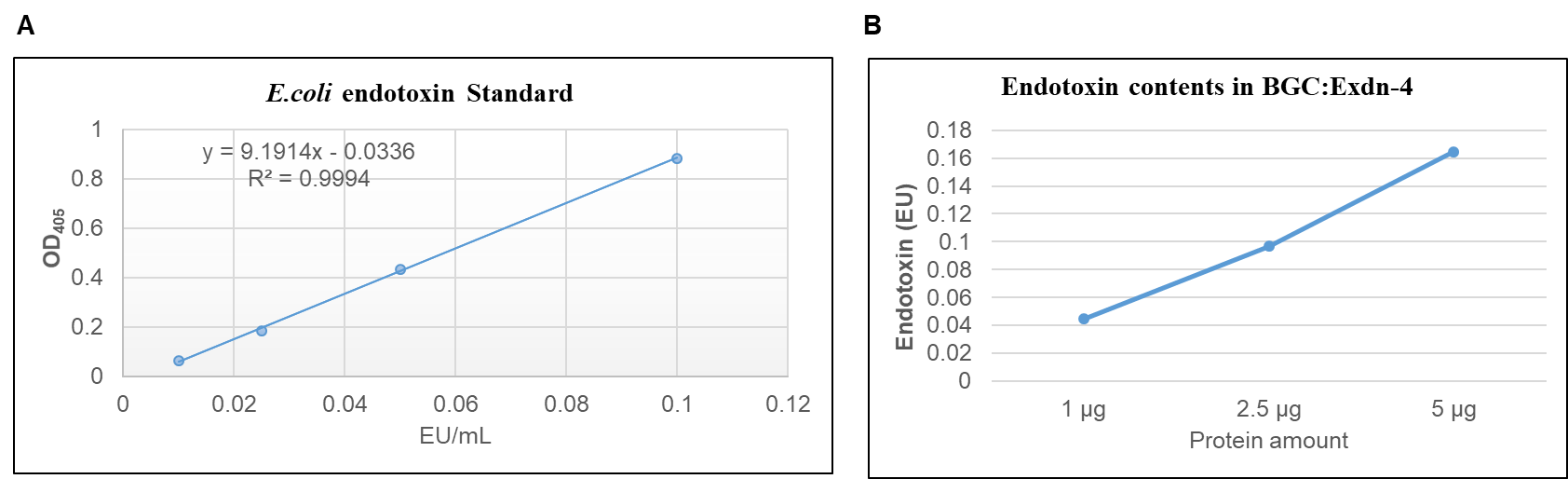
**

**Supplementary Figure 8. Plant-produced BGC:Exdn-4 contains a low level of endotoxin.**

The amount of endotoxin present in the purified BGC:Exdn-4 was measured by a chromogenic LAL assay. (**A**) The standard curve was generated using a commercial *E. coli* endotoxin standard. (**B**) Varying amounts (1–5 µg) of purified BGC:Exdn-4 were used to determine the endotoxin level. The values are means with standard deviations (n = 3).


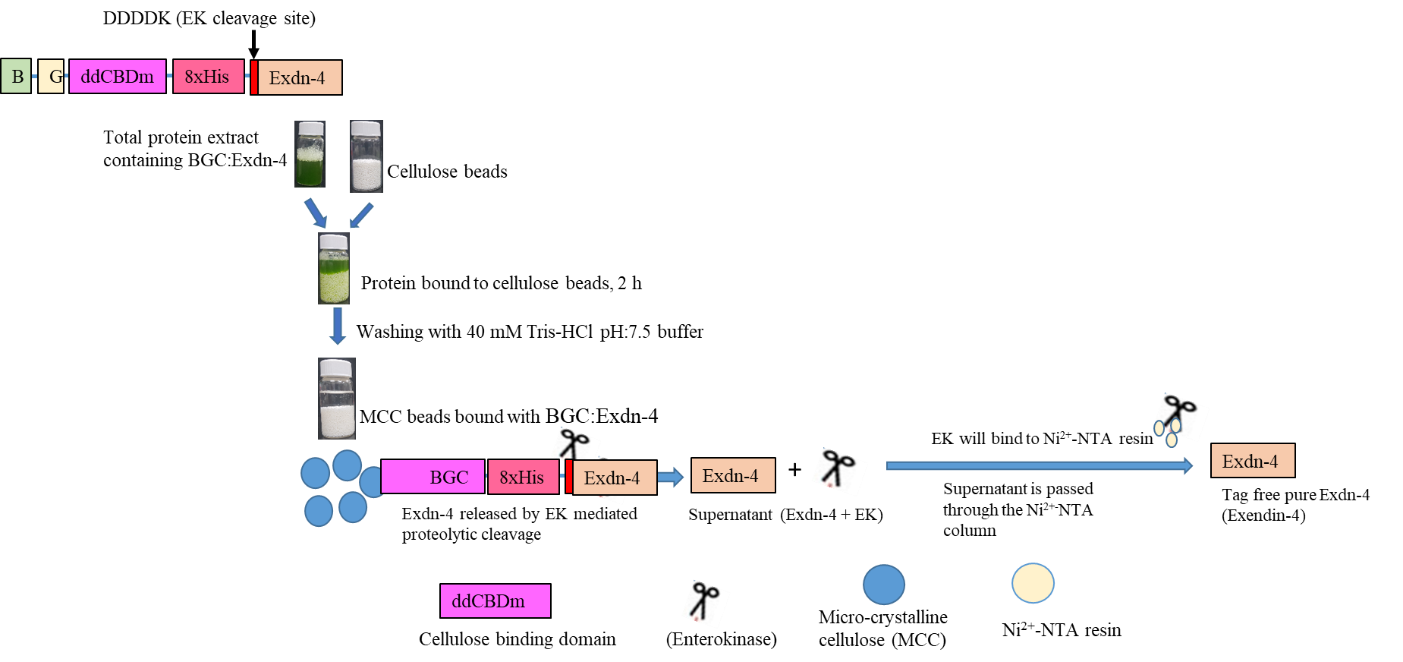


**Supplementary Figure 9. Schematic presentation for the production of tag-less Exdn-4 in *N. benthamiana.***

BGC:Exdn-4 was purified by MCC beads and processed to on-bound cleavage by enterokinase (EK). Total soluble protein extracts were mixed with MCC beads and incubated for 2 h followed by washing using washing buffer. The MCC bead-bound BGC:Exdn-4 was then treated with enterokinase, and Exdn-4 was released into the supernatant. As enterokinase contains the His tag, it was removed by Ni^2+^-NTA resin. Finally, Exdn-4 was recovered from the supernatant.

**Supplementary Table 1. Primers used in this study.**

| **Primer** | **Sequences** |
| --- | --- |
| 1. XmaI-2L-EK-F | TCCCCCCGGGGGGGCGGCGGCTCCGGCGGCGGCTCCGACGACGATGACAAG |
| 1. XhoI-HDEL-R | CCGCTCGAGCTAGAGCTCATCGTGCGATGGCGGAGGTGCCCC |
| 1. Exdn-4 qRT-F | GCATGGTGAAGGAACATTTACCAGTGACTTG |
| 1. Exdn-4 qRT-R | GAGCTACGATGGCGGAGGTGCC |
| 1. ACT qRT-F | ATGGAAACATTGTGCTCAGTG |
| 1. ACT qRT-R | GGTGCTGAGAGAAGCCAAG |

**Supplementary Table 2. Comparison of purification system used in this study**

| Purification method | **Ni^2+^-NTA** | **MCC (Microcrystalline cellulose)** |
| --- | --- | --- |
| Protein yield (final recovery) | 45.50 µg/g fresh biomass leaf | 50 µg/g fresh biomass leaf |
| Purification time (hour) | 7-8 | 3-4 |
| Column used | Yes | No needed |
| Purification strategy (binding) | Column binding | Batch binding |
| Resin (cost) | Expensive | Inexpensive |
| Affinity tag | His tag | CBD1 (Cellulose binding domain) |

**References**

Livak, K. J., and Schmittgen, T. D. (2001). Analysis of relative gene expression data using real time quantitative PCR and the 2^-ΔΔCT^ method. *Methods* 25, 402–408. doi:10.1006/meth.2001.1262.
